# Hamstring Muscle Injuries and Hamstring Specific Training in Elite Athletics (Track and Field) Athletes

**DOI:** 10.3390/ijerph191710992

**Published:** 2022-09-02

**Authors:** Pascal Edouard, Noel Pollock, Kenny Guex, Shane Kelly, Caroline Prince, Laurent Navarro, Pedro Branco, Frédéric Depiesse, Vincent Gremeaux, Karsten Hollander

**Affiliations:** 1 Inter-University Laboratory of Human Movement Biology (LIBM EA 7424), University of Lyon, University Jean Monnet, 42023 Saint Etienne, France; 2 Department of Clinical and Exercise Physiology, Sports Medicine Unit, Faculty of Medicine, University Hospital of Saint-Etienne, 42055 Saint-Etienne, France; 3 European Athletics Medical & Anti-Doping Commission, European Athletics Association (EAA), 1007 Lausanne, Switzerland; 4 Institute of Sport, Exercise and Health, University College London, London W1T 7HA, UK; 5 National Performance Institute, British Athletics, Loughborough LE11 3TU, UK; 6 School of Health Sciences (HESAV), HES-SO University of Applied Sciences and Arts Western Switzerland, 1011 Lausanne, Switzerland; 7 Department of Sprints, Hurdles and Relays, Swiss Athletics, Haus des Sports, 3063 Ittigen, Switzerland; 8 Ballet Healthcare, The Royal Ballet, London WC2E 9DA, UK; 9 Laboratoire Interuniversitaire de Biologie de la Motricité, Université Savoie Mont Blanc, EA 7424, 73000 Chambéry, France; 10 Physiotherapy Department and Motion Analysis Lab, Swiss Olympic Medical Center, La Tour Hospital, 1217 Meyrin, Switzerland; 11 Société Française des Masseurs Kinésithérapeute du Sport, SFMKS-Lab, 93380 Pierrefitte-sur-Seine, France; 12 Mines Saint-Etienne, U1059 Sainbiose, INSERM, Centre CIS, University Lyon, University Jean Monnet, 42023 Saint-Etienne, France; 13 CH Chalons en Champagne et Institut Mutualiste de Montsouris, 75014 Paris, France; 14 Swiss Olympic Medical Center, Centre de Médecine du Sport, Division de Médecine Physique et Réadaptation, Centre Hospitalier Universitaire Vaudois, 1011 Lausanne, Switzerland; 15 Institute of Sport Sciences, University of Lausanne, 1015 Lausanne, Switzerland; 16 Institute of Interdisciplinary Exercise Science and Sports Medicine, MSH Medical School Hamburg, 20457 Hamburg, Germany

**Keywords:** track and field, injury surveillance, epidemiology, risk factors, hamstring, prevention

## Abstract

Objective: We aimed to describe hamstring muscle injury (HMI) history and hamstring specific training (HST) in elite athletes. A secondary aim was to analyse the potential factors associated with in-championships HMI. Methods: We conducted a prospective cohort study to collect data before and during the 2018 European Athletics Championships. Injury and illness complaints during the month before the championship, HMI history during the entire career and the 2017–18 season, HST (strengthening, stretching, core stability, sprinting), and in-championship HMI were recorded. We calculated proportions of athletes with HMI history, we compared HST according to sex and disciplines with Chi2 tests or ANOVA, and analysed factors associated with in-championship HMI using simple model logistic regression. Results: Among the 357 included athletes, 48% reported at least one HMI during their career and 24% during the 2017–18 season. Of this latter group, 30.6% reported reduced or no participation in athletics’ training or competition at the start of the championship due to the hamstring injury. For HST, higher volumes of hamstring stretching and sprinting were reported for disciplines requiring higher running velocities (i.e., sprints, hurdles, jumps, combined events and middle distances). Five in-championship HMIs were recorded. The simple model analysis showed a lower risk of sustaining an in-championships HMI for athletes who performed more core (lumbo-pelvic) stability training (OR = 0.49 (95% CI: 0.25 to 0.89), *p* = 0.021). Conclusions: Our present study reports that HMI is a characteristic of the athletics athletes’ career, especially in disciplines involving sprinting. In these disciplines, athletes were performing higher volumes of hamstring stretching and sprinting than in other disciplines. Further studies should be conducted to better understand if and how HST are protective approaches for HMI in order to improve HMI risk reduction strategies.

## 1. Introduction

In athletics (track and field), hamstring muscle injury (HMI) represents an important challenge for athletes, health professionals, coaches and other stakeholders. HMI is one of the most prevalent injuries, especially in disciplines requiring high running velocities, with about 20% of athletes sustaining an HMI per season [1,2,3,4]. HMIs account for 17% of all injuries during international athletics championships, ranging from 0 to 35% according to sex and athletics discipline [5,6]. HMI also represents a major burden on sport practice due to time loss from sport and injury recurrence [3,4,5,6,7,8,9,10]. However, some of these studies’ results were limited by: relative small sample sizes (from 30 to 64 athletes) [1,2,3], not considering an entire athletics season and only international athletics championships (3 to 9 days in the season) [5,6], or no information on training exposure [5,6,7,8,9,10]. This justifies further studies to assess HMI epidemiology and potential risk factors, especially in elite athletes. 

Increased knowledge on athletics-specific HMI risk factors may help to improve HMI risk reduction strategies [11]. In the context of international athletics championships, male sex, older age, and disciplines requiring faster velocity have been reported to be associated with higher HMI risk [5,6]. In other athletics contexts, male sex and older age [12] were also associated with HMI, in addition to previous hamstring injury [8], lower flexibility [13] and weak or imbalanced knee flexors or hip extensors strength [1,2,3]. In contrast with other sports such as football [14,15], knowledge on factors associated with HMI risk in athletics are thus limited [1,2,3,6,8,12,13], especially in the context of elite athletes during international athletics championships [5,6].

In addition, recommendations for HMI risk reduction strategies specifically for athletics do not currently exist. Since the physical, mechanical, technical and psychological demands are different in athletics compared to football and other sports, it may be inappropriate to extrapolate results from other sports to athletics, especially elite athletes. Given the previously reported HMI risk factors in athletics [1,2,3,6,13], we can hypothesise that hamstring muscle strengthening and stretching, and preparation/training to fast velocities, may belong to HMI risk reduction strategies specifically for athletics. Lumbo-pelvic (core) conditioning has been advocated for HMI prevention, rehabilitation and athletics performance [16], and could also be a relevant additional strategy. As a first step in the development of such recommendations, improvement in the sport-specific knowledge on current practice towards HMI risk reduction strategies during usual training (i.e., hamstring specific training) in elite athletics athletes is of importance.

Therefore, we aimed to describe HMI history and hamstring specific training in elite athletes. A secondary aim was to analyse the potential factors associated with HMI occurrence during international athletics championships.

## 2. Methods

### 2.1. Study Design and Overall Procedure

We conducted a prospective cohort study to collect data before and during the 24th European Athletics Championships in Berlin in 2018 (EOC2018, 7–12 August 2018; https://www.europeanchampionships.com/2018-berlin-glasgow (accessed on 29 August 2022)) on injury and illness complaints during the month before the Championships, HMI history during the entire career, hamstring specific training, and in-championship injury and illness including in-championship HMI. There was no patient and public involvement. The study protocol was reviewed and approved by the Saint-Etienne University Hospital Ethics Committee (Institutional Review Board: IORG0007394; IRBN742020/CHUSTE) and all included athletes provided their informed consent.

### 2.2. Population

One month before the championships, European Athletics (EA, https://www.european-athletics.com (accessed on 29 August 2022)) invited all of its 51 member’s national federations to participate in the study. European Athletics sent an email including information about the study, an information letter for athletes and a pre-participation health questionnaire (PPHQ) that should be filled in by athletes. The same information was sent to national medical teams by the EA Medical and anti-doping commission. EA and EA medical and anti-doping commission, respectively, asked national federations and national medical teams whether they accepted to participate in this study and to forward the email to their athletes selected and registered for the EOC2018. Eligible athletes were athletes registered for the EOC2018. Athletes were included in the present study if they were registered for the EOC2018, member of a national federation who accepted to participate in this study, and if they completed the PPHQ.

### 2.3. Data Collection

Before the start of the championships, athletes registered for the EOC2018 were asked by EA through their national federation and/or national medical team to fill in a PPHQ. The PPHQ was developed by three sports medicine physicians with extensive experience in athletics medicine (PE, PB and FD). It was available in paper and electronic format in English. Athletes were asked to complete the questionnaire themselves and to return it to designated desks at their hotel or the warm-up area, or to their medical team who gave it to the main investigator (PE). The PPHQ included two parts similar to that from previous published studies: [17,18] (1) athletes’ characteristics: country, sex, date of birth, discipline, height, weight and mean training time per week during the four preceding weeks; (2) pre-participation health problems (analysed on a binary basis: yes/no) during the four weeks preceding the championship; and a third part (3) on history of HMI and hamstring specific training (Supplementary File S1). The last part included three questions about history of HMI: HMI during athlete’s career (yes/no), HMI during the current 2017–18 season—from October 2017 to August 2018 (yes/no), and, if so, if the respective athlete had any difficulties to participate in normal training and competition due to hamstring pain (yes/no); and then four questions about athletes’ current practice towards HMI risk reduction strategies during their usual training (i.e., hamstring specific training). These current training practice questions included four main domains of modifiable intrinsic HMI risk factors reported in athletics [1,2,3,6,13] and other sports: [15,19] hamstring strengthening, hamstring stretching, core stability (lumbo-pelvic) conditioning and/or sprint running at maximal intensity/velocity. There were no more details for athletes than the four terms presented above. For each of these four questions, athletes selected one of the five possible responses according to the frequency of that type of exercise: no (0), less than one time a month (1), more than one time a month but less than one time a week (2), more than one time a week but less than three time a week (3), more than three time a week (4) (Supplementary File S1). For the descriptive analysis and comparison according to sex and discipline we used this categorical variable (i.e., 0, 1, 2, 3 and 4) to create a hamstring specific training (HST) score by summing up the results of the four questions.

During the period of the championships, newly incurred injuries and illnesses were recorded by national medical teams (physicians and/or physiotherapists) and/or by physicians on the local organizing committee (LOC), using the same definitions and procedures than during previous international athletics championships [17,18,20]. In-championship HMI was defined as an injury reported by LOC or national medical teams located at the “posterior thigh” and with “strain/muscle rupture/tear” or “muscle cramps or spasm” as a type, based on clinical examination and/or medical imaging by the national medical teams and/or by LOC physicians, such as in previous studies [5,6].

### 2.4. Confidentiality

The athletes’ sex, date of birth and nationality were used to match data from the PPHQ and the in-championship registration of injury. Information about the purpose of the study and the procedure was provided to the athletes in writing and at information desks at the athlete hotels. All athletes were free to refuse the inclusion of their in-championship injury and illness data in the interpretation. All PPHQ and injury and illness reports were stored in a locked filing cabinet and were made anonymous after the championships. The confidentiality of all information was ensured so that no individual athlete or national team could be identified.

### 2.5. Data Analysis

We performed a descriptive analysis of the included population, using number and percentages for categorical variables and mean with standard deviations (±SD) for continuous variables, calculated the number of responders for each variable, based on the data from the PPHQ and the in-championships injury and illness data collection. Analysis of the non-responders was performed by comparing the distribution of sex, age and discipline between the eligible population and the included athletes using Chi2 tests.

Given the differences in injury rates and characteristics between sex and discipline [20], all analyses were performed by using “sex x discipline” categories. There were thus 18 different categories for the two sexes and the nine disciplines. No separated analysis was performed only between sexes or only between disciplines.

We then analysed the potential differences according to sex × discipline (i) in the distribution of number of athletes with history of HMI during their career and during the 2017–18 season using Chi2 tests, and (ii) for hamstring specific training using Chi2 tests for hamstring strengthening, hamstring stretching, core stability conditioning and/or sprinting (i.e., categorical variables) and using an ANOVA for the HST score. The significance level was initially set at *p* < 0.05. Statistical analyses were performed using JASP (JASP Team software, Version 0.14.1, University of Amsterdam, Amsterdam, The Netherlands, https://jasp-stats.org/download/ (accessed on 29 August 2022)).

To analyse the potential factors associated with HMI occurrence during the championships, we performed binomial logistic regression with in-championships HMI (yes/no) as the dependent variable and sex x discipline, age, country, history of HMI during the career (yes/no), history of HMI during the 2017–18 season (yes/no), injury complaint (yes/no), illness complaint (yes/no), strengthening, stretching, core stability, sprinting, HST score (0–16) as independent variables. Risk indicators were presented as Odds ratios (OR) and 95% confidence intervals (95% CI) for univariate and multivariable models. In a multivariable model, we examined the adjusted OR when including all variables as independent variables and in-championship HMI as a dependent variable. The significance level was initially set at *p* < 0.05. Statistical analyses were conducted using R (version 4.0.2, © Copyright 2020 The Foundation for Statistical Computing, Vienna, Austria (Comprehensive R Archive Network, http://www.R-project.org (accessed on 29 August 2022))).

## 3. Results

### 3.1. Population

Among the 51 national federations registered at the EOC2018, 24 (47.1%) agreed to participate in the study, including a total of 794 eligible athletes (50.6% of the 1570 athletes registered at the EOC2018). Among them, 357 (45.0%) athletes (22.7% of eligible athletes) agreed to participate, returned their questionnaires, and were included in the present study. Analysis of the non-responders did not show significant differences between eligible and included athletes for the distribution of discipline but did so for sex (higher proportion of female athletes in the included athletes (53.8%) compared to the eligible population (45.3%), *p* = 0.005) and age (slightly higher proportion of athletes under 20 and older than 35 among included athletes (7.0% vs. 4.0%), *p* = 0.01). None of the included athletes refused to allow their data to be used for scientific research. The characteristics of the 357 included athletes are reported in Table 1.

### 3.2. History of Hamstring Muscle Injuries

Almost half of the included athletes (48.2%) reported at least one HMI during their career, with significant difference in the distribution according to sex × discipline (*p* = 0.004) (Table 1). About a quarter (22.1%) of the included athletes reported more than one HMI during their career.

Almost a quarter of the included athletes (23.5%) reported at least one HMI during the 2017–18 season, without significant differences in the distribution according to sex × discipline (*p* > 0.05) (Table 1). Among them, 30.6% reported reduced or no participation in athletics’ training or competition at the start of the championship due to hamstring pain, representing 8.8% of all included athletes (Table 1).

### 3.3. Hamstring Specific Training

**Figure 1:**
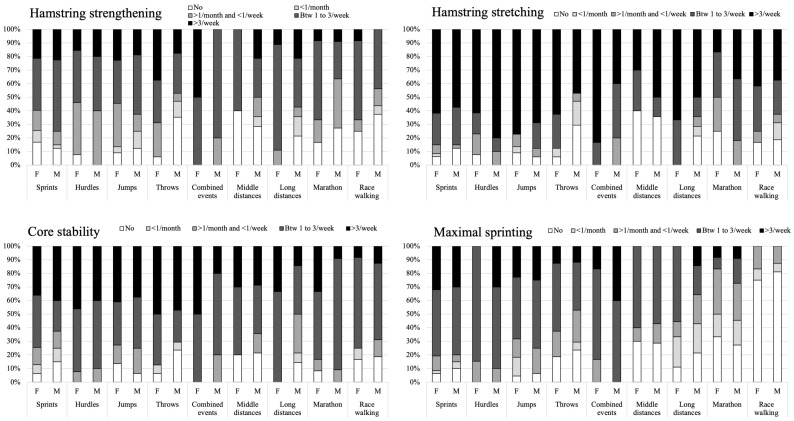
Distribution of hamstring specific training (i.e., hamstring strengthening, hamstring stretching, core stability conditioning and/or sprint running at maximal intensity/velocity) according to sex and discipline (in percentage).

**Figure 2:**
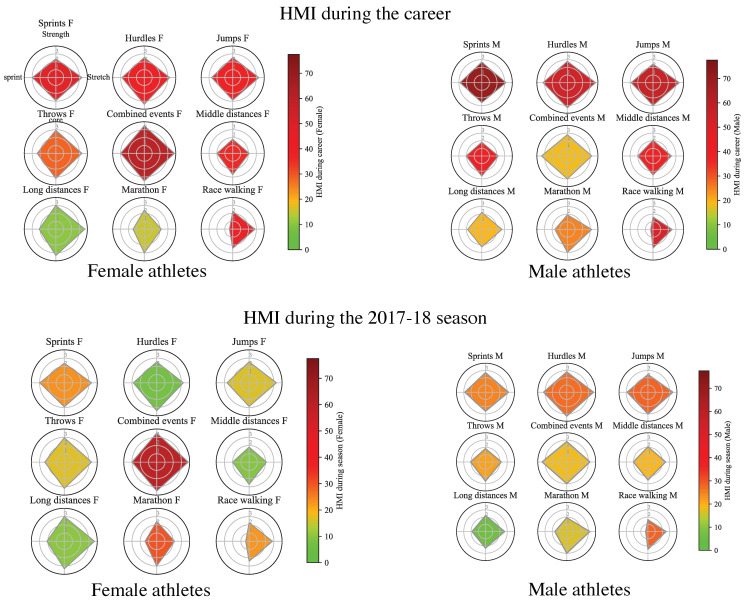
Profile of hamstring specific training according to sex and discipline, and with the history of HMI during the entire career (upper part of the figure) and during the 2017–18 season (lower part of the figure), in female (F) and male (M) athletes. The colours represent the prevalence of athletes with HMI in percentage, the targets present the mean score for athletes belonging to the sex × discipline category, for each of the four hamstring specific training types: hamstring strengthening (**up**), hamstring stretching (**right**), core stability conditioning (**down**), and sprint running at maximal intensity/velocity (**left**).

The hamstring specific training differed according to sex × discipline for hamstring stretching (Chi2 = 93.4; *p* = 0.022) and for sprint running at maximal intensity/velocity (Chi2 = 159.6; *p* < 0.001), while not for hamstring strengthening (Chi2 = 80.4; *p* = 0.145) and core (lumbo-pelvic) stability conditioning (Chi2 = 68.8; *p* = 0.449) (Table 2). Higher volumes of hamstring stretching and sprint running at maximal intensity/velocity were reported for disciplines requiring higher running velocities (i.e., sprints, hurdles, jumps, combined events and middle distances) (Figure 1 and Figure 2, and Table 2). ANOVA showed a significant effect of sex × discipline on HST score (F(17,272): 3.370; *p* < 0.001) (Table 2), with higher HST score in disciplines requiring higher running velocities.

### 3.4. In-Championship Health Problems

Among the 24 national federations who accepted to participate in this study, 16 medical teams (24%) covering 87.1% of included athletes participated in the injury and illness in-championships data collection and returned 100% of the expected report forms.

Forty-one (11.5%) of the included athletes sustained a health problem during the championships; 26 (7.3%) athletes sustained at least one in-championship injury, 17 athletes (4.7%) reported at least one in-championship illness, and 2 athletes (0.6%) sustained both.

Five in-championship HMIs were reported among the 357 included athletes during the championships (Table 1): three in male athletes (60%) and two in female athletes (40%), all in sprints (100%), three during competition (60%), and all occurring suddenly (100%).

### 3.5. Associated Factors to In-Championships Hamstring Muscle Injuries

Given the small number of athletes with in-championships HMI (*n* = 5), we only performed the simple model analysis as a descriptive analysis and results are reported in Table 3. There was a trend to a higher risk of sustaining an in-championships HMI for athletes with history of HMI during their career (OR = 4.38 (95% CI: 0.64 to 86.3), *p* = 0.189), history of HMI during the 2017–18 season (OR = 5.08 (95% CI: 0.82 to 39.2), *p* = 0.079), an injury complaint during the four preceding weeks (OR = 4.61 (95% CI: 0.75 to 35.5), *p* = 0.098), and a significant lower risk of sustaining an in-championships HMI for athletes who performed more core (lumbo-pelvic) stability training (OR = 0.49 (95% CI: 0.25 to 0.89), *p* = 0.021) (Table 3).

## 4. Discussion

The main findings of the present study were that: (1) almost half of the included athletes (48.2%) reported at least one HMI during their career, and 23.5% reported at least one HMI during the season before the championships, with a higher proportion in sprinting athletes, (2) hamstring specific training differed according to athletics sex and disciplines, and (3) some factors were associated with higher HMI occurrence during international athletics championships but should be taken with caution given the small number of reported HMI.

### 4.1. Hamstring Muscle Injuries and Athletics

In our present study, almost half of the included athletes had already experienced at least one HMI during their career. The career prevalence was as high as 72% for male sprinters. In addition, among the included athletes, about 20% reported at least one HMI during a season. This is in agreement with previous studies reporting that about 20% of athletes sustaining an HMI per season [1,2,3,4]. Specifically, these rates are 50% in female combined events athletes and 28% in male sprinters and hurdlers, which is in agreement with results from Sugiura et al. [2] and Yeung et al. [3] in sprinters. In addition, the five in-championships HMI were all in sprinters. This supports the fact that, for an athlete, the risk of sustaining a HMI during their career is high, especially in disciplines with sprint running at maximal intensity/velocity. Consequently, hamstring muscle injuries can be considered as a common injury in athletics, and especially in sprinting athletes.

The high prevalence of HMI supports the need for primary HMI risk reduction strategies, especially in sprints and disciplines requiring high running velocities. It should be noted that other disciplines such as race walking should also not been neglected (about 50% sustained at least one HMI over the career and 20–30% during the 2017–18 season). HMI risk reduction strategies have been developed and disseminated with different levels of evidence and mainly in football [21]. To our knowledge, few risk reduction suggestions have been made specifically in athletics or sprints, apart from strengthening exercises using evidence extrapolated from other sports and without scientific evaluation in athletics [8,22]. Thus, there is a need to improve knowledge on athletics-related HMI risk factors and mechanisms to develop athletics-specific risk reduction strategies, according to biomechanical requirement and injury mechanisms [6,20], and to include sprinting as part of the multifactorial HMI risk reduction [23], and scientifically evaluate their efficacy.

The high prevalence demonstrates that many competing athletes have already had a first HMI. Secondary and tertiary prevention will be needed, and efforts should continue to improve HMI management (e.g., treatment, rehabilitation, return to sport, tertiary prevention). Some rehabilitation procedures, evaluated in sprinters and jumpers through a randomized controlled trial [24], or specifically developed for HMI in athletics, can inform on current practice and encourage further work in elite athletes [10,16]. We can also take inspiration from a multifactorial criteria-based rehabilitation protocol developed and evaluated through a randomized controlled trial in football [25]. Thus, appropriate management of the first HMI is fundamental. In addition, education of athletes and their teams regarding secondary prevention (i.e., reduction of the re-injury risk) could be also a relevant part of this management [26].

### 4.2. Hamstring Specific Training

Our present results showed that athletes from disciplines requiring higher running velocities (i.e., sprints, hurdles, jumps, combined events and middle distances) performed more hamstring stretching and sprint running at maximal intensity/velocity trainings than athletes from other disciplines.

Hamstring stretching can result in increased hamstring flexibility [27]. Hamstring flexibility deficits have been identified as a weak risk factor for HMI in sprinters [13], but it is unclear whether improving flexibility reduces hamstring injury risk [27]. However, we can hypothesise that appropriate hamstring flexibility can reduce the load on the hamstring muscle, and consequently be beneficial, and not only for hamstring muscles and at long term.

Regarding sprinting training, it is logical to identify that athletes from the disciplines requiring higher running velocities are using this training modality. Most athletes performed sprinting in training 1 to 3 times per week, while 6.4% did not perform regular sprinting. Such an activity seems to be a relevant hamstring specific training modality as (i) “optimal” exposure to maximal or near-maximal running velocity is suggested as a protective HMI factor [28], and (ii) maximum sprinting activities appear to be the only way to achieve high hamstring muscle activity [29].

Hamstring strengthening seem to be performed in all disciplines without significant differences. This is consistent with the published knowledge that (i) reduced hamstring strength qualities and endurance were associated with increased HMI risk [15], and (ii) hamstring strengthening exercises were associated with reduced HMI risk [30]. However, it is important to note that 8 to 17% of athletes from disciplines with high HMI prevalence (i.e., sprints, hurdles and jumps) did not perform strengthening. This may highlight the need to improve knowledge dissemination, education or other measures in order to improve adherence to risk reduction measures [30,31].

Lumbo-pelvic (core) training has been advocated for hamstring injury prevention, rehabilitation and athletic performance [16]. In our study, lumbo-pelvic (core) training was not performed by about 1 out of 5 athletes. Although there is limited evidence to support lumbo-pelvic/core training as an integral component of training programs [32], there is rationale to support its inclusion given the role of the hamstring musculature in lumbo-pelvic stability and the likely importance of lumbo-pelvic strength in supporting hamstring function and athletic performance [33]. Improvement in core stability, lumbopelvic control, and especially better control of pelvic tilt may induce changes in sprint kinematics which allow the mechanical constraints/load on hamstring muscles during sprinting to be reduced [33], and in turn the HMI risk.

Athletes in the disciplines requiring higher running velocities had a higher HST score than athletes in other disciplines, meaning that they were using more hamstring specific training modalities. However, such athletes were also those with higher HMI prevalence. This study cannot describe causal relationships. The athletes may have completed more hamstring specific training because they experienced HMI, or because they know the high risk of HMI in their disciplines, or because such training is also of importance for sprint performance. It is also unknown from this study if the hamstring specific training increased their HMI risk. Further study is required to assess potential causal or preventative relationships between these training modalities and HMI in elite athletics athletes.

### 4.3. Strengths and Limitations

We consider a strength of this study is the population: we targeted and included elite athletes participating in international championships (i.e., European Athletics Championships in the present study), with a sample size comparable to previous studies in similar context [17,18,34]. This is the first study presenting the history of HMI and hamstring training habits in this population of elite athletics athletes, and one of the few analysing hamstring muscle injury risk factors in this sport [1,2,3,8,13].

As limitations, apart those previously discussed (i.e., recall bias, declarative bias, understandable questions) [17,18,34], the number of included athletes and of in-championships HMI collected (*n* = 5) should be highlighted. Thus, this limits the power of the statistical analyses for the secondary aim and caution should thus be taken in the interpretation and generalization of the present study’s secondary aim, which should be preferably considered as a preliminary study. Further studies should analyse the potential factors associated with HMI occurrence during international athletics championships. The response proportion to the PPHQ could be considered as relatively low (45.0% of athletes in national federations accepted to participate in this study, and 22.7% of the total targeted population). There were differences between eligible and included athletes for the distribution in sex and age. The term ‘core stability conditioning’ and other terms used in the questionnaire were not defined for athletes, that can lead to misinterpretation and underline the usefulness of athlete/patient involvement in future studies. The time frame for the hamstring specific training was not clearly defined. This was during the usual training without specifying if it was constant over the season or only during some specific period. The questionnaire was not exhaustive regarding all factors that have been reported to play a role in HMI occurrence [15,21] and in comparison to all possible suggested risk reduction approaches [35], because we tried to make it short and simple to be completed in the context of international championships. We did not evaluate technique training which may be of importance for HMI risk reduction in highly technical athletics disciplines, such as sprinting and jumping, and requires future study. We did not evaluate mechanical aspects in this study as it was a survey data collection. It is not possible to establish causal relationships between in-championships HMI and factors, however the present results help to orient future research in the field by highlighting some associations. There was unfortunately no information about exposure to the injury risk during the period of the study (i.e., hours and intensity of athletics practice). We did not analyse the influence of culture or countries on the hamstring history and specific training, but this is an area of interest for further work.

## 5. Conclusions

Our present study reports that HMI is a characteristic of the athletics athletes’ career, especially in disciplines involving sprinting. In these disciplines, athletes were performing higher volumes of hamstring stretching and sprinting than in other disciplines. Lumbo-pelvic stability training was associated with lower HMI risk in univariate analysis.

Our results support the need to improve knowledge on athletics-related HMI risk factors and mechanisms to develop athletics-specific risk reduction strategies and scientifically evaluate them. Such injury risk reduction approaches should be multifactorial considering: physical, psychological and social aspects, [31] with primary and secondary prevention approaches, including not exhaustively load management, running technique, overall health, emotional state, classical aspects (strength, flexibility, muscle strength, …), impact of fatigue, climatic conditions, and warm-up.

At elite level, injury risk reduction is at the service of performance. The introduction of injury risk reduction strategies could enable an increase in sports practice (volume and intensity) to improve performance, particularly among professional athletes, even without necessarily reducing overall risk [36]. Furthermore, given the reported negative impact of an injury on athletics performance, [37,38] and especially in championships, [39,40] the concept of health for performance could be put forward: a healthy athlete is more likely to be able to reach his/her full potential. This encourages a win-win performance-prevention approach.

## Figures and Tables

**Table 1:** Characteristics of the 357 included athletes who participated at the 24th European Athletics Championships in Berlin in 2018 and who were included in the present study, as well as their history of hamstring muscle injuries (HMI), preparticipation health problems and in-championships injuries, illnesses and hamstring muscle injuries. Data are presented using mean with standard deviations (±SD) for continuous variables and using number and frequency with percentages for categorical variables.

	Total	Sprints		Hurdles		Jumps		Throws		Combined Events		Middle Distances		Long Distances		Marathon		Race Walking	
		F	M	F	M	F	M	F	M	F	M	F	M	F	M	F	M	F	M
**Athletes’ characteristics**																			
N (%)	357 (100.0)	57 (16.0)	46 (12.9)	15 (4.2)	16 (4.5)	32 (9.0)	18 (5.0)	23 (6.4)	23 (6.4)	8 (2.2)	5 (1.4)	14 (3.9)	14 (3.9)	12 (3.4)	14 (3.9)	15 (4.2)	12 (3.4)	16 (4.5)	17 (4.8)
Age (years) (mean (SD))	26.6 (4.9)	24.8 (3.9)	25.0 (3.6)	24.1 (4.7)	24.6 (2.9)	25.1 (4.4)	26.5 (3.8)	28.3 (4.6)	28.0 (5.6)	22.2 (4.0)	25.2 (2.6)	25.7 (2.9)	25.2 (3.2)	28.7 (4.0)	26.6 (2.6)	34.6 (5.0)	32.7 (4.4)	28.7 (4.7)	28.7 (7.0)
Height (cm) (mean (SD))	177.2 (9.2)	170.5 (5.7)	182.5 (6.1)	173.0 (5.9)	186.6 (5.8)	176.7 (6.8)	187.3 (5.3)	177.2 (6.9)	189.4 (6.8)	172.9 (5.1)	185.2 (5.7)	169.3 (3.7)	181.5 (4.8)	169.0 (7.3)	178.9 (7.8)	165.8 (4.1)	179.0 (4.8)	165.8 (8.3)	179.6 (5.7)
Weight (kg) (mean (SD))	68.2 (16.7)	59.2 (5.2)	75.8 (6.2)	61.3 (5.9)	78.0 (6.7)	61.5 (6.0)	76.9 (5.7)	83.9 (14.8)	110.1 (19.2)	61.4 (3.5)	82.6 (4.4)	53.3 (3.0)	65.5 (3.6)	50.4 (5.5)	63.7 (7.6)	50.5 (4.7)	62.1 (5.4)	50.6 (5.1)	66.3 (5.2)
BMI (kg.cm^−2^) (mean (SD))	21.5 (3.8)	20.4 (1.5)	22.7 (1.4)	20.5 (1.3)	22.4 (1.3)	19.7 (1.2)	21.9 (1.3)	26.6 (3.6)	30.7 (5.5)	20.6 (1.6)	24.1 (0.8)	18.6 (1.2)	19.9 (0.8)	17.6 (0.9)	19.8 (1.2)	18.4 (1.2)	19.4 (1.4)	18.4 (0.8)	20.6 (1.4)
Mean training time per week (h) (mean (SD))	13.4 (5.1)	11.3 (3.2)	10.7 (3.6)	10.6 (4.9)	11.8 (4.0)	11.2 (4.0)	10.5 (4.8)	16.9 (4.2)	17.0 (4.7)	11.4 (2.6)	14.8 (10.3)	15.0 (5.9)	14.3 (5.7)	15.9 (2.9)	18.8 (6.1)	12.9 (3.8)	16.3 (5.9)	15.3 (2.7)	17.1 (5.8)
**History of HMI**																			
HMI during the career (*n* (%))	148 (48.2)	26 (45.6)	33 (71.7)	6 (40.0)	7 (43.8)	10 (31.3)	10 (55.6)	5 (21.7)	9 (39.1)	4 (50.0)	1 (20.0)	5 (35.7)	6 (42.9)	1 (8.3)	3 (21.4)	3 (20.0)	3 (25.0)	7 (43.8)	9 (52.9)
HMI during the 2017–18 season (*n* (%))	72 (23.5)	13 (22.8)	13 (28.3)	1 (6.7)	3 (18.8)	4 (12.5)	5 (27.8)	3 (13.0)	4 (17.4)	4 (50.0)	1 (20.0)	1 (7.1)	3 (21.4)	1 (8.3)	1 (7.1)	5 (33.3)	2 (16.7)	3 (18.8)	5 (29.4)
Reduced or no participation in athletics due to hamstring pain	27 (8.8)	4 (8.2)	3 (7.0)	0 (0.0)	1 (9.1)	1 (4.2)	2 (12.5)	2 (12.5)	2 (10.5)	2 (28.6)	0 (0.0)	0 (0.0)	1 (7.1)	1 (11.1)	2 (14.3)	2 (14.3)	1 (9.1)	1 (7.1)	2 (12.5)
**Preparticipation health problems**																			
Health problem (*n* (%))	111 (32.8)	16 (28.1)	9 (19.6)	5 (33.3)	3 (18.8)	9 (28.1)	10 (55.6)	11 (47.8)	12 (52.2)	5 (62.5)	2 (40.0)	4 (28.6)	5 (35.7)	2 (16.7)	1(7.1)	4 (26.7)	2 (16.7)	5 (31.3)	6 (35.3)
Injury (*n* (%))	103 (30.5)	15 (26.3)	8 (17.4)	5 (33.3)	3 (18.8)	9 (28.1)	10 (55.6)	10 (43.5)	11 (47.8)	5 (62.5)	2 (40.0)	3 (21.4)	3 (21.4)	2 (16.7)	1 (7.1)	4 (26.7)	2 (16.7)	4 (25.0)	6 (35.3)
Illness (*n* (%))	19 (5.6)	3 (5.3)	2 (4.3)	0 (0.0)	0 (0.0)	1 (3.1)	1 (5.6)	2 (8.7)	2 (8.7)	0 (0.0)	0 (0.0)	1 (7.1)	2 (14.3)	0 (0.0)	1 (7.1)	0 (0.0)	0 (0.0)	3 (18.8)	1 (5.9)
**In-championship health problems**																			
In-championships injuries (*n* (%))	26 (7.3)	6 (10.5)	4 (8.7)	1 (6.7)	0 (0.0)	4 (12.5)	1 (5.6)	0 (0.0)	1 (4.3)	3 (37.5)	0 (0.0)	0 (0.0)	1 (7.1)	0 (0.0)	1 (7.1)	1 (6.7)	2 (16.7)	0 (0.0)	1 (5.9)
In-championships illnesses (*n* (%))	17 (4.8)	2 (3.5)	3 (6.5)	0 (0.0)	0 (0.0)	2 (6.3)	1 (5.6)	1 (4.3)	0 (0.0)	0 (0.0)	0 (0.0)	0 (0.0)	0 (0.0)	0 (0.0)	1 (7.1)	2 (13.3)	1 (8.3)	0 (0.0)	4 (23.5)
In-championships HMI (*n* (%))	5 (1.4)	2 (3.5)	3 (6.5)	0 (0.0)	0 (0.0)	0 (0.0)	0 (0.0)	0 (0.0)	0 (0.0)	0 (0.0)	0 (0.0)	0 (0.0)	0 (0.0)	0 (0.0)	0 (0.0)	0 (0.0)	0 (0.0)	0 (0.0)	0 (0.0)

Note: Data on sex, age, discipline, height and weight were available for all responders (*n* = 357; 100%). Data on mean time training was available for 93.0% (*n* = 332), on health problem during the month before the championship for 94.7% (*n* = 338), and on history of HMI for 86.0% (*n* = 307). Percentages were calculated on the number of responders.

**Table 2:** History of hamstring muscle injuries (HMI) and hamstring specific training (HST) among the included athletes who participated at the 24th European Athletics Championships in Berlin in 2018 (*n* = 290) according to discipline and sex.

	Sprints		Hurdles		Jumps		Throws		Combined Events		Middle Distances		Long Distances		Marathon		Race Walking	
	F (*n* = 47)	M (*n* = 40)	F (*n* = 13)	M (*n* = 10)	F (*n* = 22)	M (*n* = 16)	F (*n* = 16)	M (*n* = 17)	F (*n* = 6)	M (*n* = 5)	F (*n* = 10)	M (*n* = 14)	F (*n* = 9)	M (*n* = 14)	F (*n* = 12)	M (*n* = 11)	F (*n* = 12)	M (*n* = 16)
**History of HMI**																		
HMI during the career (%)	53.2	77.5	46.2	60.0	45.5	62.5	31.3	47.1	66.7	20.0	40.0	42.9	11.1	21.4	16.7	27.3	50.0	56.3
HMI during the 2017–18 season (%)	25.5	27.5	7.7	30.0	18.2	31.3	18.8	23.5	66.7	20.0	10.0	21.4	11.1	7.1	33.3	18.2	25.0	31.3
Reduced or no participation in athletics due to hamstring pain (%)	6.4	7.5	0.0	10.0	4.5	12.5	12.5	11.8	33.3	0.0	0.0	7.1	11.1	14.3	16.7	9.1	8.3	12.5
**Hamstring Specific Training**																		
Strengthening (%)																		
No	17.0	12.5	7.7	0.0	9.1	12.5	6.3	35.3	0.0	0.0	40.0	28.6	0.0	21.4	16.7	27.3	25.0	37.5
<1/month	8.5	2.5	0.0	0.0	4.5	12.5	0.0	11.8	0.0	0.0	0.0	7.1	0.0	14.3	0.0	0.0	0.0	6.3
>1/month and <1/week	14.9	10.0	38.5	40.0	31.8	12.5	25.0	5.9	0.0	20.0	0.0	14.3	11.1	7.1	16.7	36.4	8.3	12.5
Between 1 and 3/week	38.3	52.5	38.5	40.0	31.8	43.8	31.3	29.4	50.0	80.0	60.0	28.6	77.8	35.7	58.3	27.3	58.3	43.8
>3/week	21.3	22.5	15.4	20.0	22.7	18.8	37.5	17.6	50.0	0.0	0.0	21.4	11.1	21.4	8.3	9.1	8.3	0.0
Stretching (%)																		
No	6.4	12.5	7.7	0.0	9.1	6.3	6.3	29.4	0.0	0.0	40.0	35.7	0.0	21.4	25.0	0.0	16.7	18.8
<1/month	2.1	0.0	0.0	0.0	4.5	0.0	6.3	17.6	0.0	0.0	0.0	0.0	0.0	7.1	0.0	0.0	0.0	12.5
>1/month and <1/week	6.4	2.5	15.4	10.0	9.1	6.3	0.0	5.9	0.0	20.0	0.0	0.0	0.0	7.1	25.0	18.2	8.3	6.3
Between 1 and 3/week	23.4	27.5	15.4	10.0	0.0	18.8	25.0	0.0	16.7	40.0	30.0	14.3	33.3	14.3	33.3	45.5	33.3	25.0
>3/week	61.7	57.5	61.5	80.0	77.3	68.8	62.5	47.1	83.3	40.0	30.0	50.0	66.7	50.0	16.7	36.4	41.7	37.5
Core stability (%)																		
No	6.4	15.0	0.0	0.0	13.6	6.3	6.3	23.5	0.0	0.0	20.0	21.4	0.0	14.3	8.3	0.0	16.7	18.8
<1/month	6.4	10.0	0.0	0.0	0.0	0.0	6.3	5.9	0.0	0.0	0.0	0.0	0.0	7.1	0.0	0.0	8.3	0.0
>1/month and <1/week	12.8	12.5	7.7	10.0	13.6	18.8	0.0	0.0	0.0	20.0	0.0	14.3	0.0	28.6	8.3	9.1	0.0	12.5
Between 1 and 3/week	38.3	22.5	46.2	50.0	31.8	37.5	37.5	23.5	50.0	60.0	50.0	35.7	66.7	35.7	50.0	81.8	66.7	56.3
>3/week	36.2	40.0	46.2	40.0	40.9	37.5	50.0	47.1	50.0	20.0	30.0	28.6	33.3	14.3	33.3	9.1	8.3	12.5
Sprint running at maximal intensity/velocity (%)																		
No	6.4	10.0	0.0	0.0	4.5	6.3	18.8	23.5	0.0	0.0	30.0	28.6	11.1	21.4	33.3	27.3	75.0	81.3
<1/month	2.1	5.0	0.0	0.0	13.6	0.0	0.0	5.9	0.0	0.0	0.0	0.0	22.2	21.4	16.7	18.2	8.3	6.3
>1/month and <1/week	10.6	5.0	15.4	10.0	13.6	18.8	18.8	23.5	16.7	0.0	10.0	14.3	11.1	21.4	33.3	27.3	16.7	12.5
Between 1 and 3/week	48.9	50.0	84.6	60.0	45.5	50.0	50.0	35.3	66.7	60.0	60.0	57.1	55.6	21.4	8.3	18.2	0.0	0.0
>3/week	31.9	30.0	0.0	30.0	22.7	25.0	12.5	11.8	16.7	40.0	0.0	0.0	0.0	14.3	8.3	9.1	0.0	0.0
HST score (mean (SD))	11.6 (3.5)	11.4 (4.2)	12.0 (2.6)	13.0 (1.3)	11.4 (3.5)	11.8 (3.5)	11.8 (3.7)	8.7 (5.2)	13.8 (1.6)	12.4 (1.3)	8.6 (5.2)	9.0 (5.6)	12.1 (1.9)	9.0 (4.6)	9.0 (3.4)	9.7 (3.0)	7.9 (3.9)	6.9 (3.0)

F: female athletes; M: male athletes; HMI: hamstring muscle injury; HST: hamstring specific training.

**Table 3:** Risk indicators for sustaining an in-championships hamstring muscle injury (HMI) presented as Odd Ratios (OR) with 95% confidence intervals (95% CI) calculated by univariate logistic regression analysis (*n* = 290).

	In-Championships Hamstring Muscle Injury (Univariate Model with 290 Elite Athletes)		
	*OR*	*95% CI*	*p-Value*
Sex × Discipline	1.00	0.00 to inf.	1.000
Age	1.00	0.82 to 1.18	0.967
Country	1.00	0.00 to inf.	1.000
History of HMI during the career (reference “yes”)	4.38	0.64 to 86.3	0.189
History of HMI during the 2017–18 season (reference “yes”)	5.08	0.82 to 39.2	0.079
Injury complaint	4.61	0.75 to 35.5	0.098
Illness complaint	1.00	0.00 to inf.	1.000
Strengthening	0.89	0.48 to 1.83	0.728
Stretching	0.76	0.45 to 1.39	0.319
Core stability	0.49	0.25 to 0.89	0.021
Sprint running at maximal intensity/velocity	0.94	0.50 to 1.90	0.837
HST score (0–16)	0.89	0.75 to 1.08	0.197

HMI: hamstring muscle injury; HST: hamstring specific training; OR: odds ratio; CI: confidence interval.

## Data Availability

Data are available upon reasonable request. Requests for data sharing from appropriate researchers and entities will be considered on a case-by-case basis. Interested parties should contact the corresponding author Pascal Edouard (pascal.edouard@univ-st-etienne.fr).

## Supplementary Materials

The following supporting information can be downloaded at: https://www.mdpi.com/article/10.3390/ijerph191710992/s1, Supplementary File S1: The pre-participation health questionnaire for athletes sent at the 24th European Athletics Championships in Berlin in 2018.
