# Supplementary material for: Hamstring Muscle Injuries and Hamstring Specific Training in Elite Athletics (Track and Field) Athletes
Source: Int J Environ Res Public Health. 2022 Sep 2;19(17):10992. doi: 10.3390/ijerph191710992 (PMC9518337; doi:10.3390/ijerph191710992)
Supplement: Supplementary file 1 [file ijerph-19-10992-s001.zip › ijerph-1833142-supplementary.pdf]

Supplementary material: The pre-participation health questionnaire (PPHQ).

Pre-participation health questionnaire for athletes

Dear Athlete,

In order to gain knowledge on how European Athletics can contribute to the prevention and early detection of injuries and illnesses in athletics, we would like to learn more about what **injuries or physical complaints** (such as pain, ache, stiffness, swelling, instability/giving way, locking or other symptoms) and **illnesses** you have had **in the 4 weeks prior to this championship**, even if this has not had major consequences for your participation in normal training and/or competition, and **your history about hamstring injuries and hamstring conditioning**.

All of the personal information you give on this form will be treated 100% anonymously. The compiled data and results will be published according to scientifically acceptable standards in order to help each participating nation improve their care for track and field athlete and to prevent injury. The study has received Ethical Approval by the University-Hospital of Saint-Etienne (France). We encourage you to participated by filling in the requested information and answer the following questions.

Thank you very much for your participation in this survey!

Date of birth: dd/mm/yy		Sex:	❑Female ❑ Male
Discipline:		Country:	
Height (cm):		Weight (kg):	

Average # hours of training before championships (in hours per week):	1 Week before:	2 Weeks before:	3 Weeks before:	4 Weeks before:

During the four previous weeks, have you had any difficulties participating in normal training and competition due to health problems (injury or illness)?

❑ No, full participation without health problems
❑ Yes, full participation (training or competition), but with health problems
❑ Yes, reduced participation (training or competition) due to health problems
❑ Yes, I cannot participate (training or competition) due to health problems

If you respond "Yes" and you have had health problems, could you please precise if it was (more than one response is possible):

☐ injury complaint (e.g. muscle strain, low back pain)
☐ illness (e.g. cold, infection)

Regarding your **hamstring muscles** (muscles at the posterior side of the thigh):

During your career, have you had any hamstring muscle injury?

❑ No
❑ Yes, only one
❑ Yes, more than one, if so how many:

During the 2017-18 season, have you had any hamstring muscle injury?
- ❑ No
- ❑ Yes, with no time loss in training or competition
- ❑ Yes, with less than one week of stop in training or competition
- ❑ Yes, with less than six week of stop in training or competition
- ❑ Yes, with more than six week of stop in training or competition

Currently, have you had any difficulties participating in normal training and competition due to hamstring pain?
- ❑ No, Full participation without hamstring pain
- ❑ Yes, full participation (training or competition), but with hamstring pain
- ❑ Yes, reduced participation (training or competition) due to hamstring pain
- ❑ Yes, I cannot participate (training or competition) due to hamstring pain

During your usual training, do you perform hamstring strengthening:
- ❑ No
- ❑ Yes, less than one time a month
- ❑ Yes, more than one time a month but less than one time a week
- ❑ Yes, more than one time a week but less than three time a week
- ❑ Yes, more than three time a week

During your usual training, do you perform hamstring stretching:
- ❑ No
- ❑ Yes, less than one time a month
- ❑ Yes, more than one time a month but less than one time a week
- ❑ Yes, more than one time a week but less than three time a week
- ❑ Yes, more than three time a week

During your usual training, do you perform core stability conditioning:
- ❑ No
- ❑ Yes, less than one time a month
- ❑ Yes, more than one time a month but less than one time a week
- ❑ Yes, more than one time a week but less than three time a week
- ❑ Yes, more than three time a week

During your usual training, do you perform sprint running at maximal intensity/velocity:
- ❑ No
- ❑ Yes, less than one time a month
- ❑ Yes, more than one time a month but less than one time a week
- ❑ Yes, more than one time a week but less than three time a week
- ❑ Yes, more than three time a week

Thank you very much for your participation in this survey!
